# A genome-wide assessment of genetic diversity and population structure of Korean native cattle breeds

**DOI:** 10.1186/s12863-016-0444-8

**Published:** 2016-10-20

**Authors:** Aditi Sharma, Seung-Hwan Lee, Dajeong Lim, Han-Ha Chai, Bong-Hwan Choi, Yongmin Cho

**Affiliations:** 1 Animal Genomics and Bioinformatics Division, National Institute of Animal Science, RDA, 1500, Kongjwipatjwi-ro, Iseo-myeon, Wanju-gun, Jeollabuk-do 55365 Republic of Korea; 2 Division of Animal and Dairy Science, Chugnam National University, 99 Daehak-ro, Yuseong-gu, Daejeon, 34134 Republic of Korea

**Keywords:** Hanwoo, Cattle, Korea, Genetic diversity, Illumina bovinesnp50 beadchip

## Abstract

**Background:**

The native cattle breeds are an important genetic resource for meat and milk production throughout Asia. In Asia cattle were domesticated around 10,000 years ago and in Korea cattle are being raised since 2000 B.C. There are three native breeds of cattle in Korea viz. Brown Hanwoo, Brindle Hanwoo and Jeju Black. While one of these breeds, Brown Hanwoo, is a part of a Food and Agricultural Organization and national genetic evaluation plans, others get little attention. This study is an effort to understand and provide a detailed insight into the population structure and genetic variability of the Korean cattle breeds along with other Asian breeds using various methods. In this study we report the genetic variation and structure of the Korean cattle breeds and their comparison with five other Asian cattle breeds along with a panel of animals from European *taurine*, African *taurine* and *indicine* cattle breeds.

**Results:**

Asian cattle were found to be least differentiated which reflects their recent history. Amongst the Asian breeds Hainan, which is an *indicine* breed, had the lowest gene diversity while Yanbian had the highest followed by Mongolian and Korean cattle. Amongst the Korean breeds Brown Hanwoo had the highest diversity followed by Brindle Hanwoo and Jeju Black. The genetic diversity in Asian cattle breeds was found comparable to the European *taurines* and more than the African *taurine*s and *Zebu* cattle. Korean cattle breed, Brown Hanwoo was consistently found to be closer to Yanbian, a Chinese cattle breed. We found low divergence and moderate levels of genetic diversity among the native Korean breeds. Indicine introgression from Hainan was seen in other Asian breeds. From Europe, Limousin, Holstein and Hereford introgression was found in Asian breeds.

**Conclusions:**

In this study we provide a genome-wide insight into the genetic history of the native cattle breeds of Korea. The outcomes of this study will help in prioritization and designing of the conservation plans.

**Electronic supplementary material:**

The online version of this article (doi:10.1186/s12863-016-0444-8) contains supplementary material, which is available to authorized users.

## Background

Cattle is known to be domesticated approximately 10,000 years ago near present day Turkey and Pakistan however, the earliest remains of domestic cattle found in north-east Asia dates back to only 5000 years [1]. There were multiple independent domestication events for the world cattle population as suggested by McTavish et al. [2]. Two main established splits of cattle i.e., *taurine* cattle (hump less) and *indicine* cattle (humped) are considered to have descended from aurochs which are the wild ancestors of the present day cattle breeds. Modern day breeds are a result of natural and artificial selection and adaptation to the local climate. Due to various reasons about 16 % of the cattle breeds are already extinct and 30 % face the risk of extinction. Preserving the local cattle breeds thus becomes necessary in order to prevent the depletion of the genetic resources of the country. Accessing the genetic diversity of a population provides an insight into the history, genetic structure and current status of the population which form the very basis for genetic improvement.

In North-East Asia (China, Mongolia, Korea, Japan), most of the cattle breeds are *taurine* type but China also has some *indicine/zebu* type cattle breeds and hybrid breeds (Table 1). Asian cattle are different from other *taurine* type cattle as they have been reported to have an independent mitochondrial origin [3]. Asian cattle are known to have migrated from North China to Korea via Mongolia and from Korea to Japan. Also, Korean cattle are believed to have descended as a crossbreed from European *Bos primigenius* and Indian *Bos indicus*. In Korea there are three native *taurine* type cattle breeds viz., Korean Brown cattle (BH, Hanwoo), Korean Brindle cattle (BNH, Chikso) and Jeju Black (JB, Jeju Heugu). These breeds differ from each other in the coat color and levels of nose darkness [4]. Geographically, these breeds are quite distantly located. Hanwoo is a mainland breed while Brindle Hanwoo and Jeju Black are the island breeds. All the three breeds are well adapted to the local climatic conditions. While Hanwoo can withstand temperatures as low as −20, Jeju Black shows remarkable adaptation to hot and humid climate of Jeju Island. The prevalence of brown Hanwoo is more abundant (3 million animals) as compared to the other breeds as it is the only one recognized and registered by the government for selection and breeding programs. Also, Hanwoo is believed to share its ancestor with Yanbian, a Chinese cattle breed, until the last century [5]. Like most other Asian cattle breeds the history of Korean cattle breeds too is not very well documented. There are some references of these animals to be used as draft animals and also used in religious sacrifices [6]. It was in 1979 that a government regulated breeding program called “Hanwoo-Gaeryang-Danji (HGD)” was initiated for the native Hanwoo cattle [7]. Hanwoo is mainly selected for the carcass weight and meat quality traits like Marbling , backfat thickness, Loin eye area. Owing to the government led programs Hanwoo is now one of the superior commercial livestock breeds of Korea. Recently, government has shown an increased interest towards the conservation of other breeds and also towards the development of these breeds as an alternate beef breed. Availability and cost of genome-wide Single Nucleotide Polymorphism (SNP) panel had made it a popular choice of method to accesses diversity of various livestock species [8, 9]. In this study we utilized illumina BovineSNP50 BeadChip Ver. 1 (Illumina, San Diego, CA, USA) to genotype three native Korean cattle breeds along with Yanbian resulting in 54,609 genotyped SNPs. We combined data from 11 representative breeds from the BovineHapmap dataset comprising of European *taurine*, African *taurine* and *Indicine* (*Zebu*) cattle and five Asian breeds from data published by Decker et al. [10].

**Table 1:** Breeds, breed code and number of samples used in the study

Breed name	Breed code	Sample size	Type	Meat/dairy
Brown Hanwoo	BH	120	*Taurine*	Meat
Brindle Hanwoo	BNH	20	*Taurine*	Meat
Jeju Black	JB	20	*Taurine*	Meat
Yanbian	YB	39	*Taurine*	Meat/Draught
Wagyu	WAGY	12	*Taurine*	Meat
Qinchuan	QC	4	Hybrid	Meat
Hainan	HN	4	*Indicine*	Draught
Luxi	LX	5	Hybrid	Meat
Mongolian	MG	5	*Taurine*	Meat
Angus	ANG	24	*Taurine*	Meat
Brahman	BRM	25	*Indicine*	Meat
Nelore	NEL	21	*Indicine*	Meat
Gir	GIR	24	*Indicine*	Dairy
Brown Swiss	BS	22	*Taurine*	Dairy
Hereford	HFD	21	*Taurine*	Meat
Holstein	HOL	60	*Taurine*	Dairy
Jersey	JER	49	*Taurine*	Dairy
Limousin	LMS	25	*Taurine*	Meat/Milk
N’Dama	ND	56	*Taurine*	Meat/Milk
Sheko	SHK	20	*Taurine*	Meat/Draught

In our study we present a detailed insight into the genetic diversity and structure of Korean cattle breeds in comparison with Chinese, Mongolian and Japanese breeds. The outcomes of this study would shed light on the genomic structure of Korean breeds as well as other Asian breeds and this information could be used as a primer to design conservation strategies and breeding programs.

## Methods

### Animals and genotyping

Blood samples were collected from Brown Hanwoo (BH), Brindle Hanwoo (BNH) and Jeju Black (JB). All three breeds are found in their native track in three different regions of the country. Breeds and number of samples used for the study are described in detail in Table 1. BH is a mainland breed while BNH and JB are island breeds. Utmost care was taken to avoid any crossbreds during sampling. YB (Chinese cattle) samples were made available by Dr. Lee SH from Chungnam National University in Daejon, South Korea. Genotyping data for the Asian cattle breeds was downloaded from dryad.org [11]. European *taurine*, African *taurine* and Zebu data was used from the Bovine Hapmap project. All the genotype data was then merged to make one final dataset. In the final dataset there were 576 samples and 35,598 SNPs.

Genomic DNA for genotyping assays was extracted from the blood sample using DNeasy 96 Blood and Tissue Kit (Qiagen, Valencia, CA, USA). DNA quantification was performed using a NanoDrop 1000 (Thermo Fisher Scientific Inc., Wilmington, DE, USA). DNA samples were submitted for genotyping with total DNA of 900 ng, 260/280 ratio >1.8, and DNA concentration of 20 ng/ul. The genotyping for animals was done by the Animal Genome & Bioinformatics Division of the National Institute of Animal Science, RDA, Korea, using a BovineSNP50 BeadChip Ver.1. (Illumina, San Diego, CA, USA).

### Quality control of the SNP data

Genotype data was imputed using Beagle program [12]. Plink version1.09 (http://pngu.mgh.harvard.edu/purcell/plink/) [13]) was used for the quality control of the raw genotype data. After merging all the genotype data we had a total of 35,598 SNPs. SNP genotypes were subjected to filtering based on Minor Allele Frequency (MAF) >0. 001, Hardy Weinberg Equilibrium <1E-06 and genotype frequency (0.05). Ten thousand nine hundred twenty-five markers were removed based on Hardy Weinberg test, 1664 markers were removed based on genotype rate, 842 markers were removed based on MAF. After Quality control the final dataset consisted of 22,672 SNPs. A total of 576 samples were analyzed in the study.

### Genetic diversity and population differentiation analysis

To understand the genetic diversity of the cattle populations we used Hierfstat R package [14]. Genetic similarities between breeds were accessed with their pairwise Fst values. The Fst values describes the difference in allele frequencies between two independent populations with a potential value of 0 to 1, with 1 being the most different/ distantly related. Fst-distance matrix was then used for the hierarchical clustering of the breeds. Poppr R package [15] was used to calculate the Provesti’s absolute genetic distances between populations and further compute a Neighbor joining tree.

### Population structure

Population structure of the Korean cattle breeds was studied using multivariate approach and model based methods. Multi-dimensional scaling (MDS) was used to capture the preliminary glimpse of the genetic structure of the Korean cattle populations and also to remove outliers, if any. MDS (Additional file 1: Figure S1) was computed using Plink version1.09 and plotted in R version 3.2.2 (R Development Core Team, 2008). Principal component Analysis (PCA) and Discriminant analysis of Principal components (DAPC) was performed for the genetic clustering of individuals and breeds. Adegenet [16] R package was used for the PCA and DAPC analysis. We retained 100 principal components for DAPC which explained ~52 % of the total variance of the data.

Unsupervised hierarchical clustering was performed using the Admixture 1.23 software [17]. Admixture performs maximum likelihood estimation of individual ancestries from multilocus SNP genotype datasets. An in-house R script was then used to plot the ancestry of individuals of different breeds. While plotting, individuals were ordered according to the fraction ancestry they shared with other individuals. And as each individual shared a different proportion of ancestry with different individuals of different breeds, not necessarily all the individuals of the same breed grouped together in the final plots.

Patterns of population splits and mixtures in the history of the populations were studied using Treemix [18] program. Allele frequencies in the 20 cattle populations were used to infer the structure.

## Results

### Genetic diversity and differentiation

The genetic diversity within populations was accessed as a measure of heterozygosity i.e., expected heterozygosity (Hs) and observed heterozygosity (Ho). Gene diversity in three Korean cattle breeds was found to be slightly less (0.257) than the European *taurine* cattle breeds (0.274) and higher than the zebu cattle breeds (0.162). YB cattle breed had Hs = 0.271 while the Korean native breeds including BH, BNH and JB had Hs value of 0.257 (Table 2). Amongst the Asian breeds HN, which is an *indicine* breed, had the lowest gene diversity while YB had the highest followed by MG and Korean cattle. Amongst the Korean breeds BH had the highest diversity followed by BNH and JB. Fis was used to quantify for the non-random mating (Inbreeding) and we found less inbreeding than expected as Fis values were negative for all the breeds ranging from −0.16 in BRM to −0.23in HN. Among all the three native breeds BNH had the lowest Fis value. The negative Fis values averaged over all the loci indicated a lack of population structure in the populations. Given the small population size of native Korean breeds (BNH = 4000 and JB = 1000) considerable diversity still exists in the native populations.

**Table 2:** Genetic diversity in the 20 cattle breeds as measured using Hs, Ho and Fis

S. No	Breed	Expected heterozygosity (Hs)	Observed heterozygosity (Ho)	Inbreeding (Fis)
1	Brown Hanwoo	0.2625	0.3352	−0.2100
2	Brindle Hanwoo	0.2551	0.3310	−0.2298
3	Jeju Black	0.2546	0.3268	−0.2156
4	Yanbian	0.2710	0.3437	−0.2048
5	Wagyu	0.2340	0.3039	−0.2203
6	Qinchuan	0.2515	0.3162	−0.1999
7	Hainan	0.1374	0.1795	−0.2348
8	Luxi	0.2156	0.2687	−0.1749
9	Mongolian	0.2649	0.3317	−0.1894
10	Angus	0.2798	0.3611	−0.2227
11	Brahman	0.1764	0.2225	−0.1607
12	Nelore	0.1542	0.2019	−0.1990
13	Gir	0.1546	0.2005	−0.2297
14	Brown Swiss	0.2553	0.3342	−0.2314
15	Hereford	0.2930	0.3806	−0.2297
16	Holstein	0.2875	0.3711	−0.2286
17	Jersey	0.2505	0.3234	−0.2167
18	Limousin	0.2801	0.3582	−0.2146
19	N’Dama	0.2106	0.2721	−0.2114
20	Sheko	0.2087	0.2663	−0.2013

Hs is expected heterozygosity; Ho is Observed heterozygosity; Fis is the inbreeding coefficient

Genetic differentiation between the 20 populations was studied using pairwise Fst estimates (Additional file 2: Table S1). Genetic differentiation between both BH and BNH and BH and JB was 0.02 while between BH and YB, QC, LX, MG it was 0.01. Amongst the Korean native breeds Fst value between BNH and JB were the highest (0.06). Korean breeds had the lowest pairwise Fst values compared to other *taurine* and zebu breeds. Amongst the three native breeds the Fst estimates of BH with other European *taurine* breeds were the least. The island breeds BNH and JB were found to be more differentiated from the *taurine* and zebu breeds. On an average, the genetic differentiation between the Asian breeds was found to be less than the other breeds in the study.

Provesti’s genetic distances between populations were calculated using adegenet R package and were plotted as a neighbor joining tree (Additional file 1: Figure S2). The results corroborate well with the Fst analysis. Three distinct groups viz. European *taurine*, Asian *taurine* and Zebu were observed. Korean cattle along with Japanese WAGY and Chinese YB cattle formed a separate group apart from European and African *taurines*. BNH and BH culminated on the same node. Due to geographical proximity YB is believed to be closely connected to BH until the Korean War [19]. HN, LX, QC, SHK and ND clubbed with zebu cattle while MG formed a group with European *taurine* cattle. The Korean cattle cluster was found between the European *taurine* on one side and Zebu on the other. It reflects the influence of European *taurine* and Zebu cattle on the present day Korean and Japanese cattle breeds. BH was found to be more closely related to YB (0.097), followed by BNH (0.143) and JB (0.142). Amongst the three native breeds, BNH and JB were found to be most distantly related than others (0.176). Genetic distances of Korean breeds with that of other breeds were the least with LMS (0.22) and the highest with HN (0.32). Compared to other Asian breeds genetic distance of BH from WAGY was found to be the smallest (0.202).

### Population structure

**Fig. 1:**
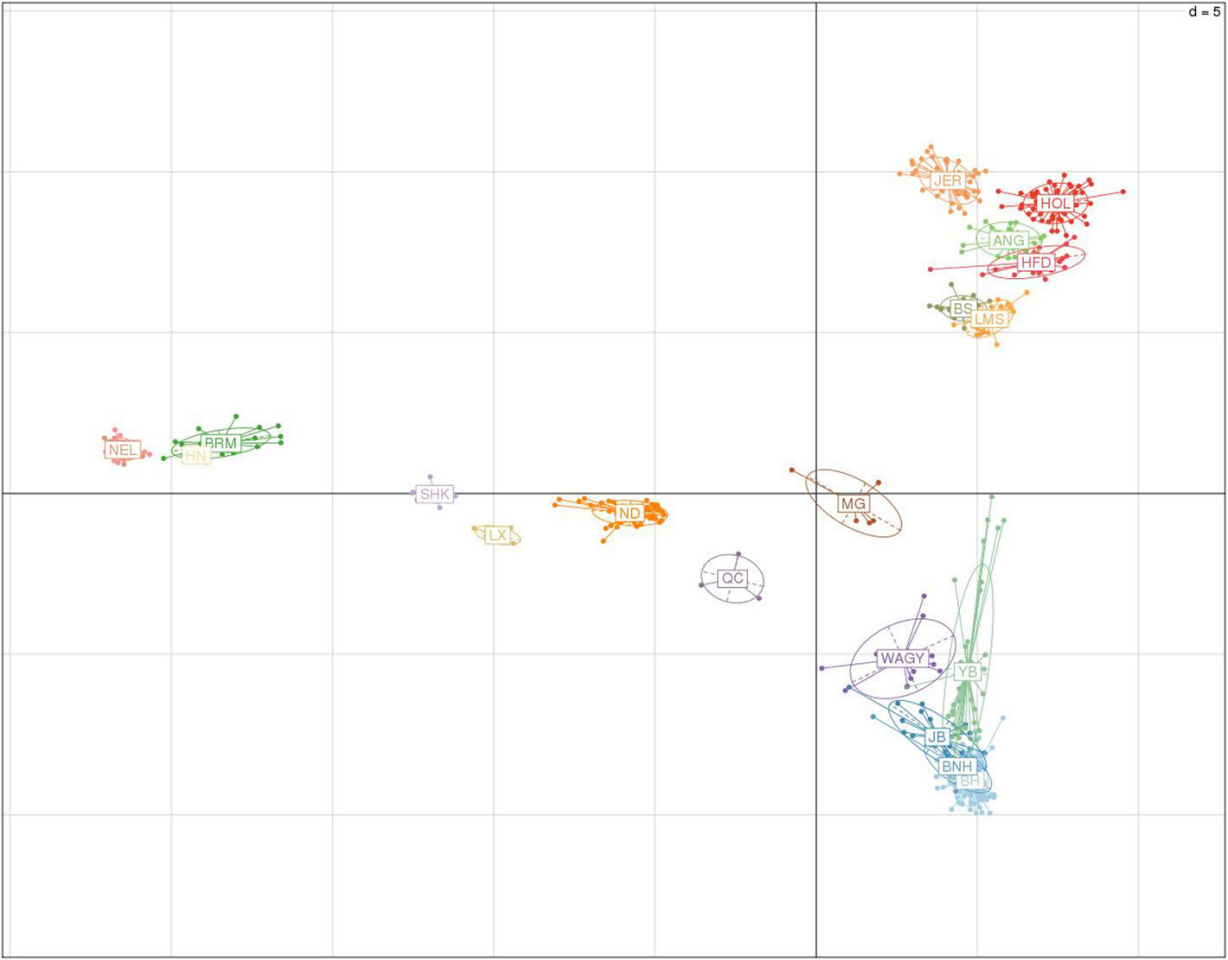
PCA plot of the 20 cattle breeds plotted using 576 total animals and 22,672 SNPs spread across the genome

Population structure of the Korean cattle breeds was studied using multivariate approach and model based methods. Principal component Analysis (PCA) was used to place the Korean Cattle breeds with respect to the European *taurine*, African *taurine* and Zebu. PCA is particularly important as it is a powerful method to capture the variation in the genotypic dataset. In our study PCA grouped the individuals in one cluster depending on the origin of population. We observed 20 clearly separated clusters (Fig. 1). The first component split the data according to *taurine/indicine* split and the second component divided the data according to African/European *taurine* split. BH, BNH, JB and YB individuals formed their individual clusters and due to genetic relatedness the clusters were formed almost overlapping with each other. Along with the closeness of Korean breeds amongst themselves they also showed closeness to WAGY. Other Asian cattle breeds lied in between the zebu and Korean cattle cluster. MG cattle formed a cluster close to Korean and Japanese cattle. In our analysis, Korean breeds formed the most compact cluster compared to other breeds known to share same ancestry. SHK (Ethiopian cattle breed), LX, ND and QC placed themselves center to all the major clusters i.e. zebu, European *taurine* and African *taurine* cluster. This shows the influence of European and *indicine* cattle breeds on the formation of these Asian and African breeds.

We then performed the DAPC analysis. DAPC method transforms the data using PCA and then uses the discriminant analysis to identify the clusters. In our analysis 100 PCs were retained which explained ~52 % variation. Results obtained from the DAPC analysis corroborated with those obtained from PCA analysis. Individuals were correctly assigned to their respective clusters. We initially started this study with a total of 21 populations. The 21^thst^ population used in the study was a local Korean cattle breed called “Chosun”. But based on DAPC, PCA and MDS results we understood that these animals were only crossbreds so we removed the entire population from the analysis and used only 20 populations in this study (Additional file 1: Figure S3).

### Model-based population structure

**Fig. 2:**
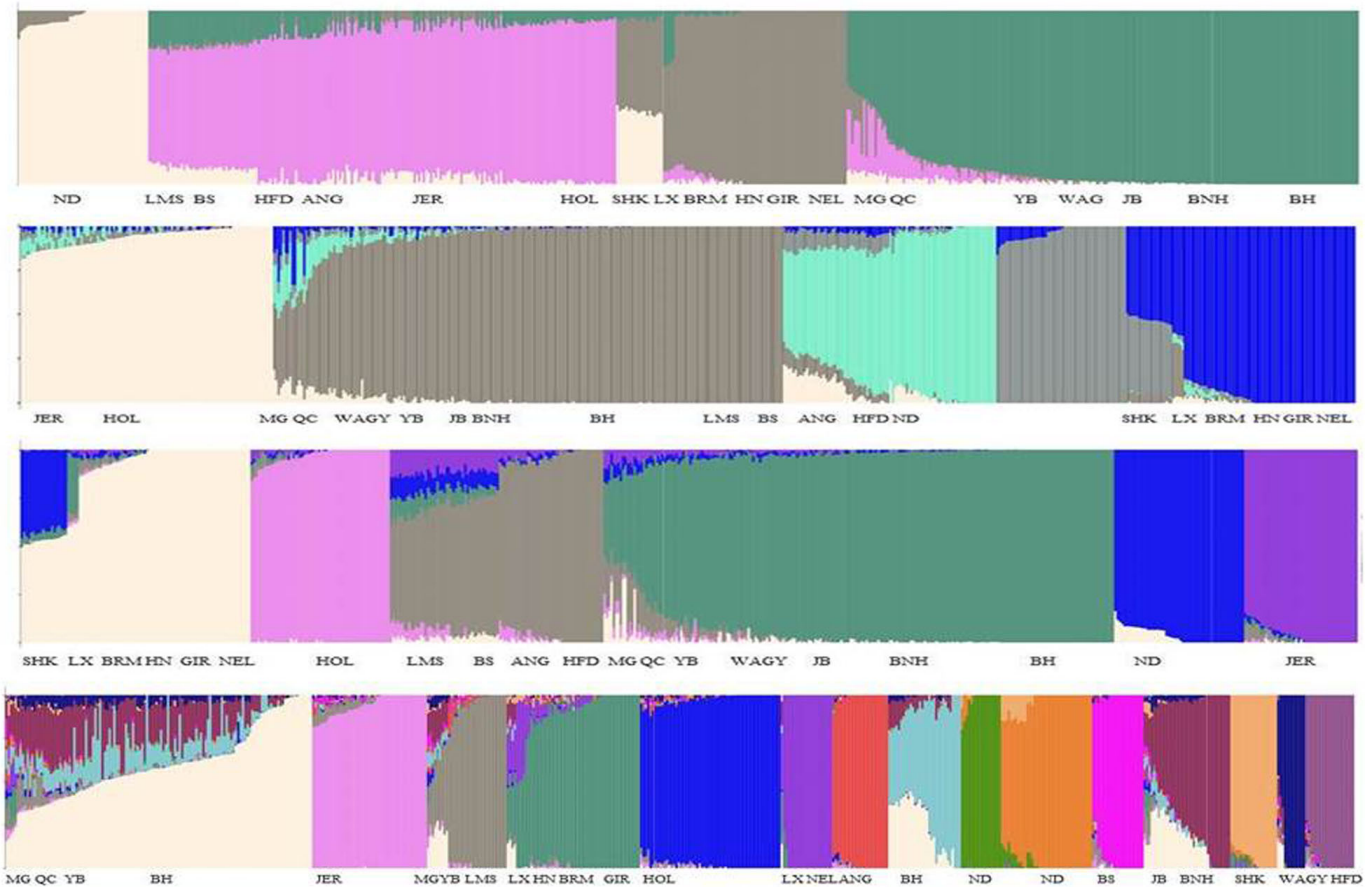
Unsupervised clustering of individuals plotted according to fraction ancestry in 20 populations. From top to bottom, K = 4, K = 5, K = 6 and K = 15. In the plots not necessarily all the individuals grouped together. Sometimes individuals formed different groups based on the genetic composition. Therefore, same breed name could be seen two or more times in the same plot

We performed unsupervised hierarchal clustering of our data as implemented in Admixture software . Admixture estimates the ancestry in a model-based manner from the autosomal SNP panel from a set of unrelated individuals. The data was then plotted in order of fraction ancestry that they shared with other breeds (Fig. 2). In our study at K = 3, we observed separation of European, Zebu and Asian -African *taurine* cattle breeds. At K = 3 Asian and African N’Dama cattle formed one group together. Asian cattle breeds formed a separate group from N’Dama at K = 4. Variable proportion of admixture from Zebu, European and African breeds were observed in Asian breeds. At K = 4, most of the BH animals showed little or no admixture from any other breeds. At K = 6, LX, which is a hybrid and HN, which is zebu, formed a group with BRM, GIR and NEL. SHK and LX shared ~50–60 % ancestry with zebu breeds. SHK was formed mainly from Zebu and N’Dama but it also showed some admixture with Asian breeds. LX, was formed from Zebu and Asian breeds with some admixture from European and African cattle. Asian breeds didn’t differentiate into separate breeds until K = 10. At K = 10, WAGY separated as a separate breed while other Asian breeds (Except LX and HN) formed one group together. The optimum value of K was found to be K = 15 (Additional file 1: Figure S4) and at this value of K the Asian breeds started to show differentiation. While some of the individuals of BH, BNH and JB made two different groups, most of them still clubbed together. YB individuals formed a group with BH at almost all values of K. The Asian *taurines* differentiated well from the African and European *taurines*, however some European nad African introgression was still seen in the Asian cattle breeds. Along with the European and African admixture Korean breeds showed admixture amongst themselves too. A group of YB animals were found to share ~20 % ancestry with LMS. MG and QC cattle were found to have multiple ancestries. YB shared a large proportion (~50–60 %) of ancestry with BH and ~10 % each with BNH and JB. Both island breeds BNH and JB were found to have BH admixture in them. Admixture of some fraction of African cattle was observed in Asian cattle breeds.

Treemix analysis was used to study the population splits and gene flow (Fig. 1). We first constructed a phylogenetic tree without adding any migration events followed by adding upto 8 migration events for value. Without any migration events we saw all the 20 populations divide into two major groups i.e. *Taurine* and *Indicine*. Within *taurine*, Korean cattle breeds along with Japanese WAGY and Chinese YB formed a separate group. HN and LX formed a group with indicine cattle breeds. As we added migration events we found influence of European cattle, LMS on both Asian and African cattle. When adding more migration evnts than 8 we found introgression from HOL and HFD into the Asian cattle breeds. Introgression of *Indicine* genetic component from HN was seen in the Asian breeds. We also found influence of MG on the Chinese LX and QC breeds.

## Discussion

In our analysis, diversity was assessed as a measure of expected heterozygosity (Hs) and observed heterozygosity (Ho). Diversity in Korean cattle breeds was found to be more than Zebu and African *Taurine* and less than European *Taurine*. This could be attributed to the recent genetic history of Korean cattle breeds. However, genetic diversity of Korean cattle breeds in our study was considerably lower than that reported by Kim et al. [20]. This might be because Kim et al. used microsatellite markers for the analysis. Also the average observed heterozygosity value for Korean cattle in our study was found to be 0.33 while Edea et al. [9], based on 8 k Illumina SNP chip, reported Ho value to be 0.41. In both the studies observed heterozygosity value was found to be higher in Korean cattle breeds than the African breeds. The observed heterozygosity values in our study for Korean breeds were similar to that observed by Strucken et al. [21]. Within the Korean breeds observed heterozygosity was found to be considerably higher than the expected heterozygosity. Observed heterozygosity values were found to be least in JB followed by BNH. This could be attributed to their lower population size as reported by Choi et al. [22]. However, despite the small population size the observed heterozygosity values were not remarkably different from the mainland breed BH.

In Korea only BH has a dedicated breeding program and the number of animals is ~3 million while number of animals for JB and BNH is only a few thousands. We used Fis as a measure to study inbreeding within these populations. Fis values indicated an excess of heterozygotes in BNH (−0.230) and JB (−0.216) which are the island populations. Compared to BH (−0.210), YB (−0.205) was found to be less inbred. The Fis values in our study were different from that reported by Choi et al. [5]. This could be because of the use of different type of data (microsatellite markers) for the calculations. Korea follows a 20 KPN system in the breeding program. In this program 20 superior bulls are used for artificial insemination across the country. So, despite a good population size of around three million, BH is found to be more inbred than other Korean populations. Given the population sizes, selection strategies and implementation of designed breeding programs elevated Fis was expected in this domestic cattle breed. Inbreeding in Korean populations on an average was similar to the European *taurine* cattle breeds. Zebu breeds were found to be least inbred amongst these 15 populations.

Pairwise Fst was used to study the population differentiation between the twenty breeds in the study. The Fst values for Korean cattle breeds in our study were lower than the European breeds ranging from 0.02 and 0.06. YB and BH were found to be least differentiated from each other (Fst = 0.01). JB and BNH were found to be most differentiated from one another (Fst = 0.06) while they were found to be less differentiated from BH which is a mainland breed. The divergence of JB and BNH in two different directions from the mainland breed makes a good example of the classical island model. Lower Fst values in the Korean populations suggest that they had not yet differentiated well into completely separate/independent breeds. While BH was found to be closer to MG, LX, HN and QC and WAGY (0.02), BNH and JB were found to be more differentiated from other Asian breeds. Based on Fst values, we found that Asian breeds were still genetically closer to each other than the European, African *taurine* and Zebu cattle.

**Fig. 3:**
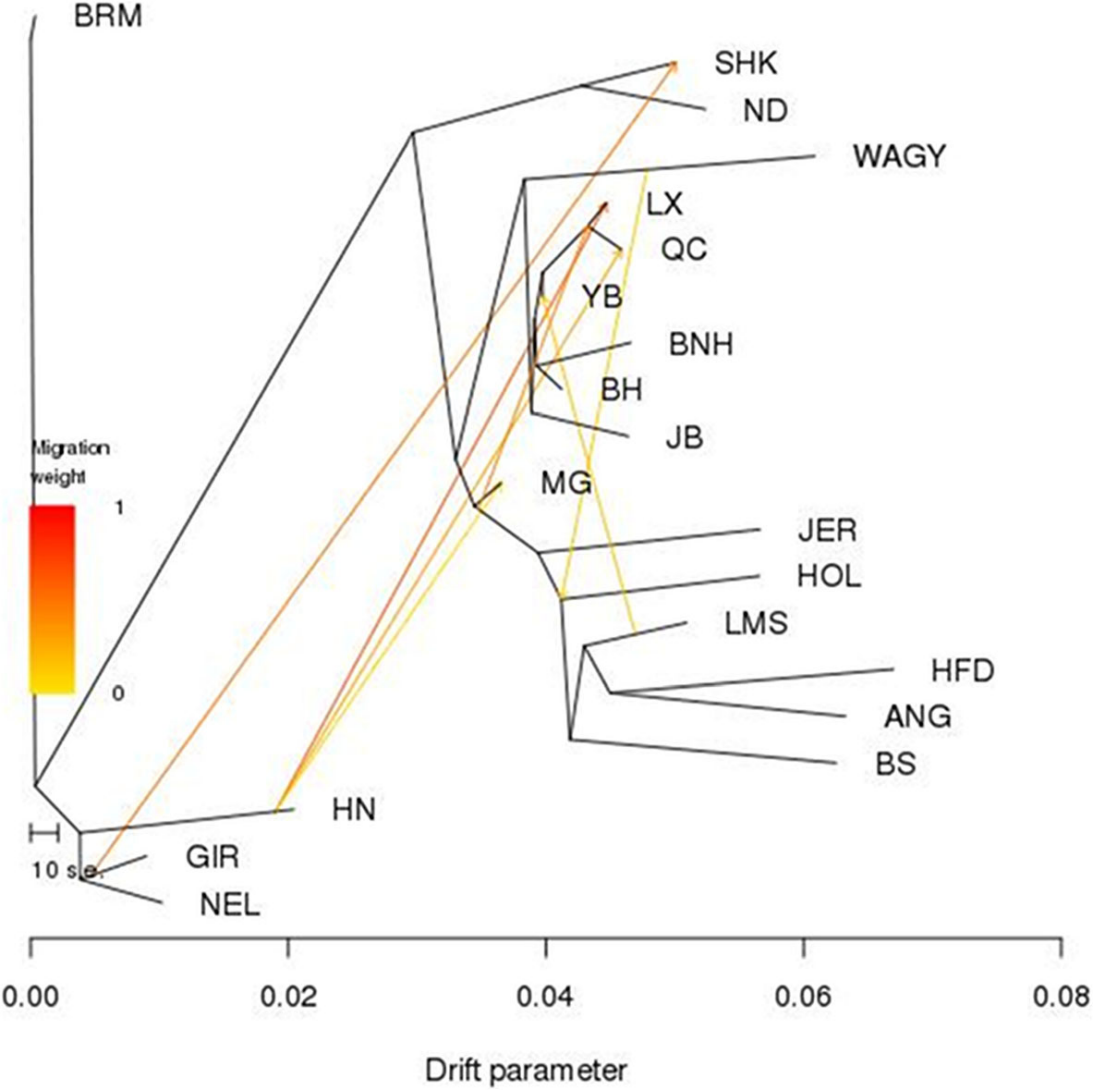
Maximum likelihood tree inferred from 20 cattle breeds without any migration edges. Brahman (BRM) was used as an outgroup. The scale bar depicts ten times the average standard error of the estimated entries in the sample covariance matrix

We also found evidence of an admixed lineage of Korean cattle breeds. Influence from European *taurine* and *Indicine* cattle in varying proportions were observed in the Korean cattle breeds. Genetic influence of Korean cattle breeds from LMS and BS was nicely captured by PCA and DAPC plots. Admixture analysis (Fig. 2) at all the values of K starting from K = 3 showed small proportion of admixture of Korean cattle with African cattle. However, based on treemix analysis (Fig. 3) there was no evidence of direct gene flow between African and Asian breeds. We found the introgression of HN in MG, LX and QC. Gene flow from European cattle breeds LMS, HFD and HOL was also seen in Asian cattle. Based on various population metrics used in the study BH was consistently found to be closer to YB than the other native breeds. The differences in the breeds could be attributed to the selective breeding of BH. Our study also showed an island effect on the JB and BNH. The genetic diversity in Korean cattle breeds was found comparable to the European *taurines*.

## Conclusion

Modern day Asian cattle are a result of introgression from various European and Indicine breeds. Mongolian, Qinchuan and Yanbian cattle still contain a high level of admixture from various other breeds while present day Korean cattle were seen to be less admixed. While Korean cattle were found to be less admixed with the breeds outside Korea, they still were found to be admixed amongst themselves. Based on all the population metrics used to study genetic diversity we conclude that the Korean populations are still very closely related and have not yet differentiated enough to be considered as separate breeds. These breeds could rather be referred to as the subpopulations of BH. YB was found to be closest to BH and thus it could be developed as an alternate meat breed of the country. Since YB is an unselected population it can also serve as a model to study the effect of selection and breeding on BH. BNH and JB are two valuable genetic resources of the country and we suggest relevant measures to be taken to increase the number of individuals in the two island breeds and thus prevent the loss of diversity that may occur due to small population size.

## Additional files

Additional file 1: Figure S1. MDS plot of the 20 breeds. Each color in the plot corresponds to the color in the legend. **Figure S2.** Provesti’s genetic distances based Neighbor-Joining tree of 20 cattle breeds. Tree is clearly divided into three groups - one group consists of the Zebu and composite cattle, one consists of European *taurine* and one group is formed entirely of Asian breeds, mostly Korean. **Figure S3.** DAPC plot of four Korean breeds. This includes Chosun cattle (CS) breed which was later removed from the study. **Figure S4.** Optimum value of k for the admixture analysis. (DOCX 233 kb)
Additional file 2: Table S1. Pairwise Fst values between 20 populations used in the study. **Table S2.** Absolute genetics distance or Provesti’s distance between the 20 cattle populations used in the study. (DOCX 26 kb)

## Abbreviations

ANG: Angus
B.C: Before Christ
BH: Brown Hanwoo
BNH: Brindle Hanwoo
BRM: Brahman
BS: Brown Swiss
DAPC: Discriminant analysis of principal components
DNA: Deoxyribonucleic acid
GIR: Gir
HF: Hereford
HGD: Hanwoo-Gaeryang-Danji
Ho: Observed heterozygosity
HOL: Holstein
Hs: Expected heterozygosity
JB: Jeju Black
JER: Jersey
KPN: Korea proven bull number
LMS: Limousine
MAF: Minor allele frequency
MDS: Multi-dimensional scaling
ND: N’Dama
NEL: Nelore
ng/ul: Nanogram Per microlitre
PCA: Principal components analysis
SHK: Sheko
SNP: Single nucleotide polymorphism
YB: Yanbian
