# Erratum to: A genome-wide assessment of genetic diversity and population structure of Korean native cattle breeds

**DOI:** 10.1186/s12863-017-0472-z

**Published:** 2017-02-10

**Authors:** Aditi Sharma, Seung-Hwan Lee, Dajeong Lim, Han-Ha Chai, Bong-Hwan Choi, Yongmin Cho

**Affiliations:** 1 Division of Animal Genetics and Bioinformatics, National Institute of Animal Science, RDA, 1500, Kongjwipatjwi-ro, Iseo-myeon, Wanju-gun, Jeollabuk-do 55365 Republic of Korea; 2 Division of Animal and Dairy Science, Chugnam National University, 99 Daehak-ro, Yuseong-gu, Daejeon, 34134 Republic of Korea

## Erratum

The original article [1] has been updated because the previous version was not the most recent one. This is due to a mistake in production and not the fault of any authors.
