# Supplementary material for: A genome-wide assessment of genetic diversity and population structure of Korean native cattle breeds
Source: BMC Genet. 2016 Oct 20;17:139. doi: 10.1186/s12863-016-0444-8 (PMC5072310; doi:10.1186/s12863-016-0444-8)
Supplement: Additional file 1: Figure S1. — MDS plot of the 20 breeds. Each color in the plot corresponds to the color in the legend. Figure S2. Provesti’s genetic distances based Neighbor-Joining tree of 20 cattle breeds. Tree is clearly divided into three groups - one group consists of the Zebu and composite cattle, one consists of European taurine and one group is formed entirely of Asian breeds, mostly Korean. Figure S3. DAPC plot of four Korean breeds. This includes Chosun cattle (CS) breed which was later removed from the study. Figure S4. Optimum value of k for the admixture analysis. (DOCX 233 kb) [file 12863_2016_444_MOESM1_ESM.docx]

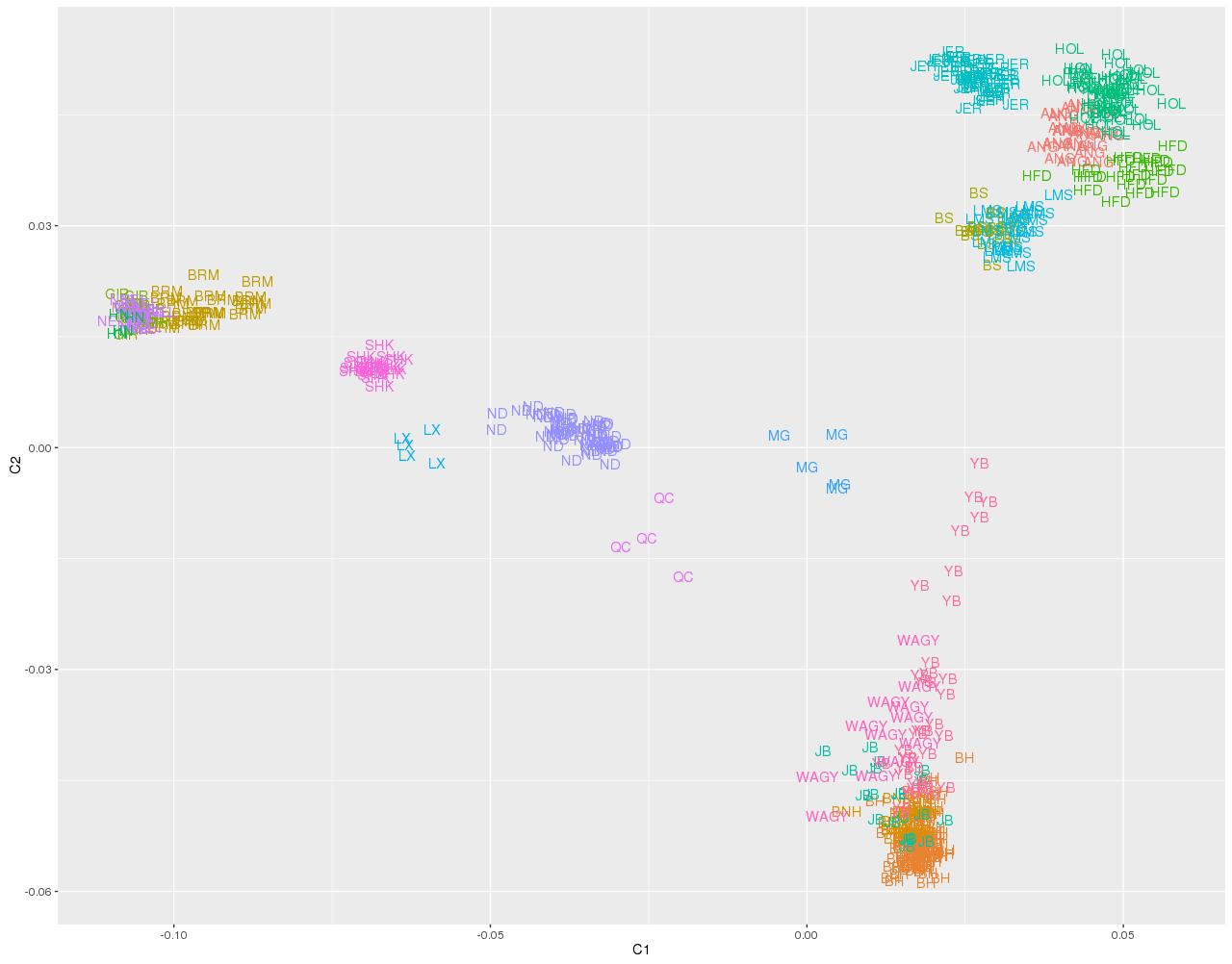


**Figure S1**: MDS plot of the 20 breeds. Each breed is represented by its breed code and a different color.
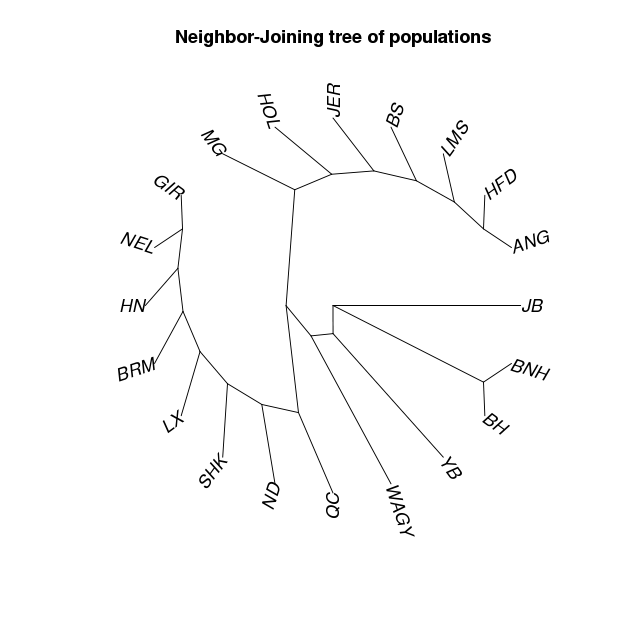


**Figure S2**: Provesti’s genetic distances based Neighbor-Joining tree of 20 cattle breeds . Tree is clearly divided into three groups - one group consists of the Zebu and composite cattle, one consists of European taurine and one group is formed entirely of Asian breeds, mostly Korean.


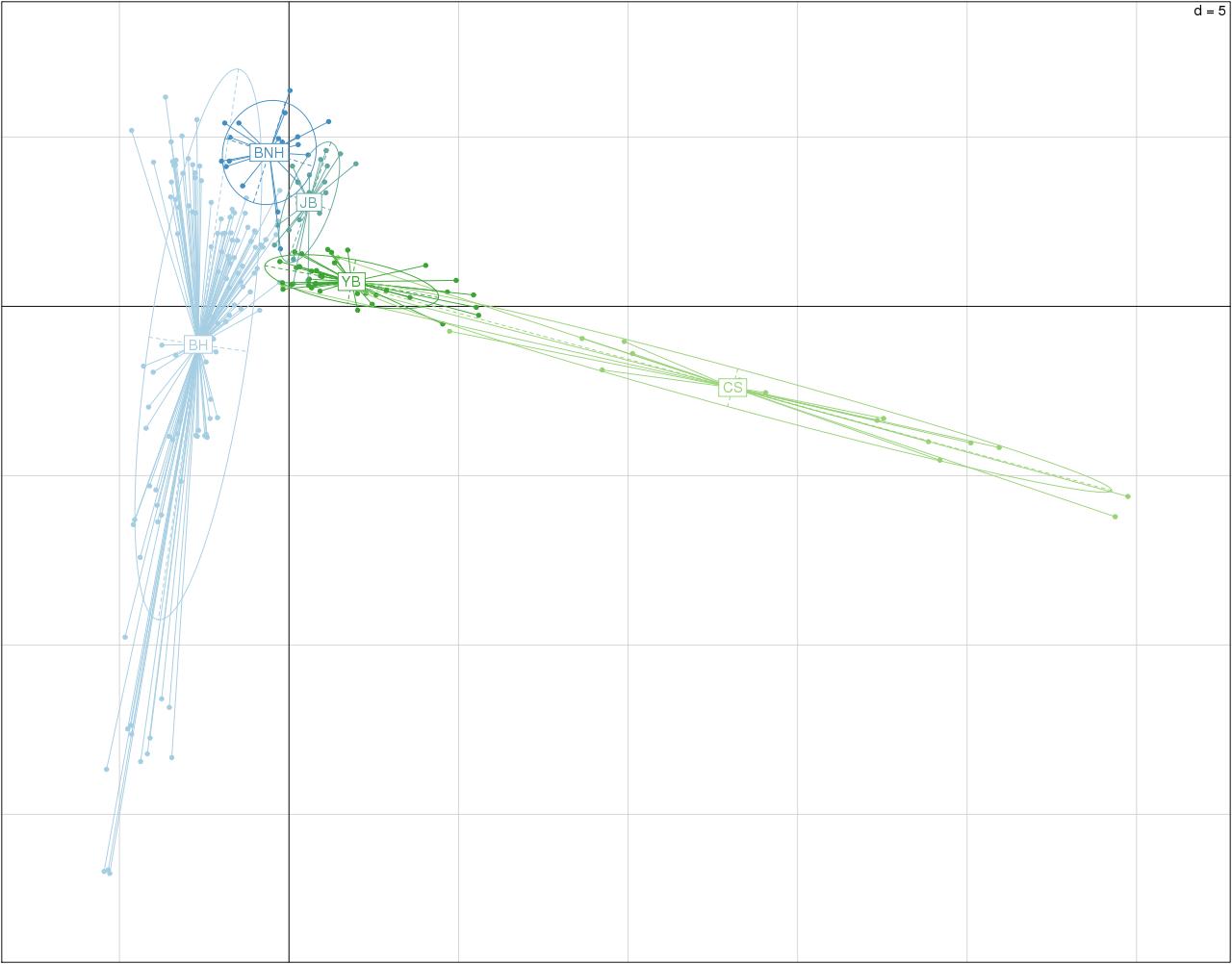


**Figure S3**. DAPC plot of Korean cattle breeds. This includes Chosun cattle (CS) breed which was later removed from the study.


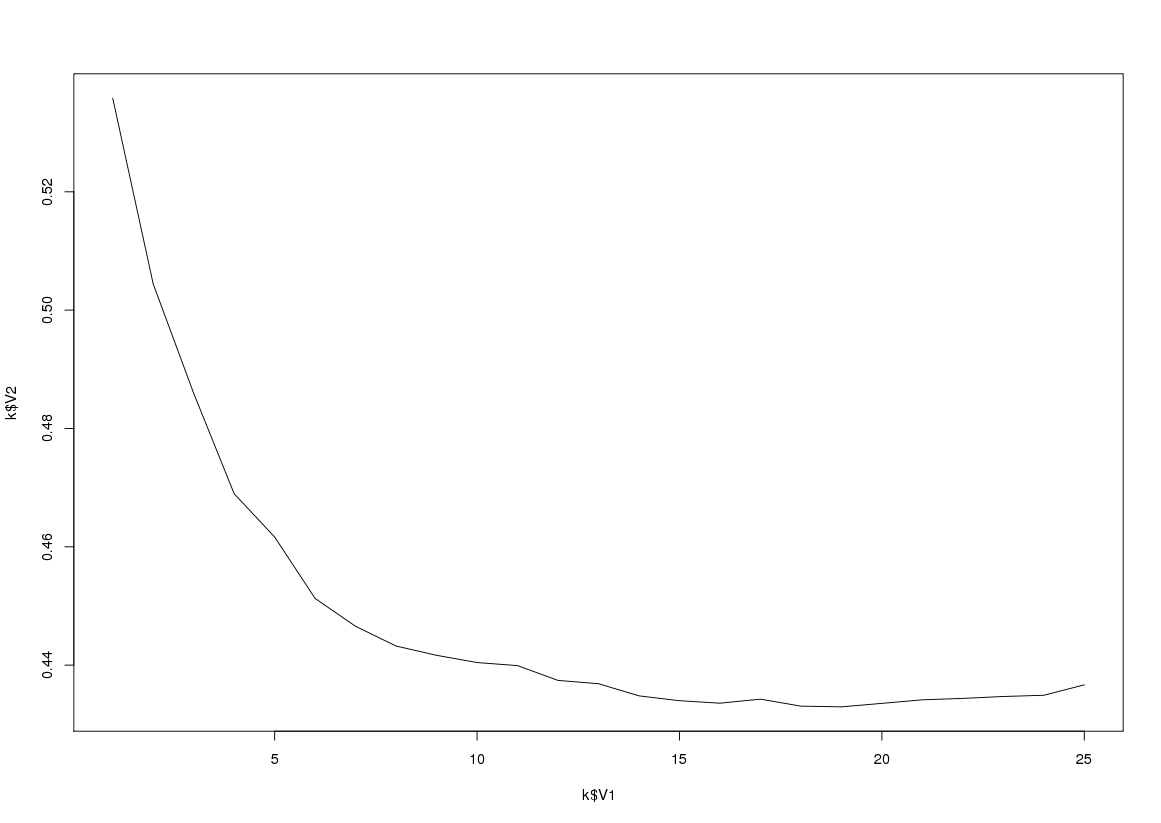


**Figure S4**: Optimum value of K for the admixture analysis
