# Supplementary material for: A genome-wide assessment of genetic diversity and population structure of Korean native cattle breeds
Source: BMC Genet. 2016 Oct 20;17:139. doi: 10.1186/s12863-016-0444-8 (PMC5072310; doi:10.1186/s12863-016-0444-8)
Supplement: Additional file 2: Table S1. — Pairwise Fst values between 20 populations used in the study. Table S2. Absolute genetics distance or Provesti’s distance between the 20 cattle populations used in the study. (DOCX 26 kb) [file 12863_2016_444_MOESM2_ESM.docx]

|  | **BH** | **BNH** | **JB** | **YB** | **ANG** | **BRM** | **BS** | **GIR** | **HFD** | **HOL** | **JER** | **LMS** | **ND** | **NEL** | **SHK** | **WAGY** | **QC** | **HN** | **LX** |
| --- | --- | --- | --- | --- | --- | --- | --- | --- | --- | --- | --- | --- | --- | --- | --- | --- | --- | --- | --- |
| **BNH** | 0.02 |  |  |  |  |  |  |  |  |  |  |  |  |  |  |  |  |  |  |
| **JB** | 0.02 | 0.06 |  |  |  |  |  |  |  |  |  |  |  |  |  |  |  |  |  |
| **YB** | 0.01 | 0.03 | 0.03 |  |  |  |  |  |  |  |  |  |  |  |  |  |  |  |  |
| **ANG** | 0.05 | 0.11 | 0.10 | 0.07 |  |  |  |  |  |  |  |  |  |  |  |  |  |  |  |
| **BRM** | 0.06 | 0.14 | 0.13 | 0.10 | 0.15 |  |  |  |  |  |  |  |  |  |  |  |  |  |  |
| **BS** | 0.05 | 0.12 | 0.11 | 0.07 | 0.11 | 0.16 |  |  |  |  |  |  |  |  |  |  |  |  |  |
| **GIR** | 0.07 | 0.17 | 0.16 | 0.12 | 0.18 | 0.05 | 0.19 |  |  |  |  |  |  |  |  |  |  |  |  |
| **HFD** | 0.05 | 0.11 | 0.11 | 0.08 | 0.10 | 0.16 | 0.12 | 0.19 |  |  |  |  |  |  |  |  |  |  |  |
| **HOL** | 0.06 | 0.06 | 0.06 | 0.06 | 0.07 | 0.11 | 0.07 | 0.12 | 0.07 |  |  |  |  |  |  |  |  |  |  |
| **JER** | 0.06 | 0.08 | 0.08 | 0.07 | 0.09 | 0.12 | 0.09 | 0.15 | 0.09 | 0.08 |  |  |  |  |  |  |  |  |  |
| **LMS** | 0.04 | 0.08 | 0.08 | 0.05 | 0.07 | 0.13 | 0.08 | 0.16 | 0.08 | 0.05 | 0.07 |  |  |  |  |  |  |  |  |
| **ND** | 0.08 | 0.10 | 0.09 | 0.09 | 0.12 | 0.12 | 0.12 | 0.14 | 0.12 | 0.11 | 0.12 | 0.10 |  |  |  |  |  |  |  |
| **NEL** | 0.07 | 0.18 | 0.17 | 0.12 | 0.18 | 0.06 | 0.20 | 0.07 | 0.19 | 0.12 | 0.14 | 0.16 | 0.13 |  |  |  |  |  |  |
| **SHK** | 0.04 | 0.11 | 0.11 | 0.07 | 0.13 | 0.09 | 0.14 | 0.11 | 0.14 | 0.08 | 0.10 | 0.11 | 0.07 | 0.11 |  |  |  |  |  |
| **WAGY** | 0.02 | 0.06 | 0.06 | 0.04 | 0.09 | 0.12 | 0.10 | 0.14 | 0.10 | 0.05 | 0.07 | 0.07 | 0.07 | 0.15 | 0.10 |  |  |  |  |
| **QC** | 0.01 | 0.05 | 0.05 | 0.02 | 0.06 | 0.06 | 0.07 | 0.08 | 0.07 | 0.02 | 0.03 | 0.05 | 0.03 | 0.09 | 0.06 | 0.13 |  |  |  |
| **HN** | 0.02 | 0.10 | 0.10 | 0.05 | 0.09 | 0.04 | 0.11 | 0.05 | 0.10 | 0.04 | 0.05 | 0.08 | 0.05 | 0.06 | 0.07 | 0.19 | 0.25 |  |  |
| **LX** | 0.01 | 0.07 | 0.06 | 0.03 | 0.07 | 0.04 | 0.08 | 0.06 | 0.08 | 0.03 | 0.04 | 0.06 | 0.04 | 0.06 | 0.05 | 0.14 | 0.15 | 0.17 |  |
| **MG** | 0.01 | 0.05 | 0.05 | 0.02 | 0.05 | 0.07 | 0.06 | 0.10 | 0.06 | 0.02 | 0.03 | 0.04 | 0.04 | 0.11 | 0.07 | 0.12 | 0.15 | 0.24 | 0.15 |

**TableS1**. Pairwise Fst values between 20 populations used in the study.

**Table S2.** Absolute genetics distance or Provesti 's distance between the 20 cattle populations used in the study.

|  | BH | BNH | JB | YB | ANG | BRM | BS | GIR | HFD | HOL | JER | LMS | ND | NEL | SHK | WAGY | QC | HN | LX |
| --- | --- | --- | --- | --- | --- | --- | --- | --- | --- | --- | --- | --- | --- | --- | --- | --- | --- | --- | --- |
| BNH | 0.143 |  |  |  |  |  |  |  |  |  |  |  |  |  |  |  |  |  |  |
| JB | 0.142 | 0.176 |  |  |  |  |  |  |  |  |  |  |  |  |  |  |  |  |  |
| YB | 0.097 | 0.150 | 0.148 |  |  |  |  |  |  |  |  |  |  |  |  |  |  |  |  |
| ANG | 0.233 | 0.261 | 0.256 | 0.225 |  |  |  |  |  |  |  |  |  |  |  |  |  |  |  |
| BRM | 0.245 | 0.262 | 0.256 | 0.246 | 0.298 |  |  |  |  |  |  |  |  |  |  |  |  |  |  |
| BS | 0.227 | 0.253 | 0.250 | 0.220 | 0.259 | 0.285 |  |  |  |  |  |  |  |  |  |  |  |  |  |
| GIR | 0.267 | 0.280 | 0.276 | 0.271 | 0.320 | 0.118 | 0.304 |  |  |  |  |  |  |  |  |  |  |  |  |
| HFD | 0.248 | 0.277 | 0.272 | 0.241 | 0.263 | 0.320 | 0.278 | 0.343 |  |  |  |  |  |  |  |  |  |  |  |
| HOL | 0.204 | 0.238 | 0.233 | 0.196 | 0.235 | 0.287 | 0.246 | 0.311 | 0.257 |  |  |  |  |  |  |  |  |  |  |
| JER | 0.213 | 0.240 | 0.236 | 0.207 | 0.244 | 0.269 | 0.241 | 0.289 | 0.264 | 0.230 |  |  |  |  |  |  |  |  |  |
| LMS | 0.196 | 0.229 | 0.225 | 0.179 | 0.221 | 0.275 | 0.220 | 0.299 | 0.240 | 0.209 | 0.215 |  |  |  |  |  |  |  |  |
| ND | 0.219 | 0.243 | 0.238 | 0.219 | 0.280 | 0.245 | 0.266 | 0.257 | 0.300 | 0.263 | 0.253 | 0.246 |  |  |  |  |  |  |  |
| NEL | 0.272 | 0.285 | 0.279 | 0.275 | 0.325 | 0.129 | 0.306 | 0.122 | 0.347 | 0.315 | 0.293 | 0.302 | 0.260 |  |  |  |  |  |  |
| SHK | 0.217 | 0.237 | 0.235 | 0.217 | 0.279 | 0.177 | 0.266 | 0.185 | 0.302 | 0.262 | 0.252 | 0.248 | 0.184 | 0.190 |  |  |  |  |  |
| WAGY | 0.202 | 0.226 | 0.224 | 0.205 | 0.281 | 0.274 | 0.272 | 0.289 | 0.300 | 0.266 | 0.264 | 0.256 | 0.263 | 0.292 | 0.260 |  |  |  |  |
| QC | 0.224 | 0.244 | 0.243 | 0.224 | 0.296 | 0.248 | 0.281 | 0.260 | 0.315 | 0.280 | 0.275 | 0.267 | 0.266 | 0.264 | 0.243 | 0.268 |  |  |  |
| HN | 0.311 | 0.319 | 0.317 | 0.314 | 0.358 | 0.192 | 0.339 | 0.180 | 0.381 | 0.352 | 0.326 | 0.340 | 0.295 | 0.184 | 0.239 | 0.315 | 0.272 |  |  |
| LX | 0.233 | 0.252 | 0.249 | 0.234 | 0.300 | 0.199 | 0.286 | 0.206 | 0.319 | 0.285 | 0.275 | 0.273 | 0.259 | 0.210 | 0.217 | 0.270 | 0.235 | 0.202 |  |
| MG | 0.210 | 0.235 | 0.231 | 0.207 | 0.269 | 0.262 | 0.259 | 0.278 | 0.288 | 0.252 | 0.252 | 0.239 | 0.259 | 0.282 | 0.246 | 0.259 | 0.255 | 0.309 | 0.248 |
